# Sensitivity of Field Tests for Assessment of Wrestlers Specific Fitness

**DOI:** 10.2478/hukin-2022-0069

**Published:** 2022-09-08

**Authors:** Milan Marković, Lazar Toskić, Filip Kukić, Ivan Zarić, Milivoj Dopsaj

**Affiliations:** 1 Faculty of Sport and Physical Education, University in Priština – Kosovska Mitrovica, Leposavić, Serbia; 2 Faculty of Sport, University ‘’Union – Nikola Tesla’’, Belgrade, Serbia; 3 Physical Fitness Testing and Research, Police Sports Education Center, Abu Dhabi Police, Abu Dhabi UAE; 4 Faculty of Sport and Physical Education, University of Belgrade, Belgrade, Serbia; 5 Institute of Sport, Tourism and Service, South Ural State University, Chelyabinsk, Russia

**Keywords:** combat sports, wrestling, testing, discriminative

## Abstract

The aim of this study was to determine the differences in performance, cardiac, and metabolic variables of combat-specific fitness of wrestlers from varying sports levels. The sample consisted of 61 active and highly trained adult wrestlers of national and international levels from Serbia, from both Greco-Roman and freestyle forms of wrestling, allocated into three groups: national team wrestlers (N = 15), first league competitive wrestlers (N = 20), and second league competitive wrestlers (N = 26). Participants performed two tests: a Specific Wrestling Fitness Test (SWFT) and a Specific Wrestling Performance Test (SWPT). Between-group differences were determined using multiple analysis of variance (MANOVA). Significant differences were obtained between wrestlers of different sports levels in Tn^Throws^ (F = 77.491, p < 0.001), SJF^Index^ (F = 49.710, p < 0.001), and SWP^Index^ (F = 31.205, p < 0.001) in the SWFT and in Tn^Throws^ (F = 71.051, p < 0.001), SJF^Index^ F = 45.343, p < 0.001), and SWP^Index^ (F = 26.820, p < 0.001) in the SWPT. Based on the results, it could be concluded that the SWFT and the SWPT provide very good sensitivity in the evaluation of the wrestlers specific fitness of wrestlers.

## Introduction

Testing is typically used to assess the physical abilities and/or physical performance of athletes, while the obtained results are used to set individually adjusted training loads and to control and monitor progress (Franchini et al., 2007; Hübner-Woźniak et al., 2006; Lidor et al., 2006). Testing in sports is a complex problem as it needs to determine multiple factors that influence performance and how these factors interact. As wrestling belongs to the category of sports in which open and closed motor patterns are combined, i.e., a group of acyclic-semi-structural sports, it is very difficult to organise testing of wrestlers under combat-specific conditions. This includes active situational resistance that depends on the level of physical, technical and tactical preparedness of the opponent, which cannot be controlled.

Although laboratory tests are scientifically valid, reliable and sensitive, they often insufficiently reproduce the real situational sports-competitive loads. To that end, there is a growing need for the development of specific field tests which represent the close approximation of sports activity in which the time structure, intensity, metabolic and functional efforts of the fight can be monitored and measured (Maglischo, 2003; Müller et al., 2000). Specific tests for testing sports performance are required to have high validity, reliability and sensitivity, but also situational specificity (Hopkins et al., 2001; Utter et al., 1997; Wilson and Murphy, 1996). In martial arts, judokas have shown the greatest interest in these types of tests. Through a large number of published papers, various types of specific tests in judo have been published, the most popular test being the “Specific Judo Fitness Test” (Detanico and Santos, 2012; Franchini et al., 1998; Hesari et al., 2014). Although similar tests are not absent in wrestling (Curby, 2005; Shiyan, 2011; Starosta and Tracewski, 1998; Starosta and Baić, 2015), none of the abovementioned tests have fully established the basic metric characteristics which would qualify a test or a measuring instrument for measurement purposes.

In the absence of the necessary tests that could be used in scientific research, as well as in practice for the needs of diagnosis and selection in wrestling, Marković et al. (2017) created two specific field tests for wrestlers (a Specific Wrestling Fitness Test [SWFT] and a Specific Wrestling Performance Test [SWPT]). The SWFT was developed to mimic the physical and metabolic loads of wrestling combat, while the SWPT additionally mimics the structure of combat as per the current rules. While the SWPT showed higher validity and could be recommended for periodical assessments, the SWFT is simpler, easier to administer and could be performed within a training session for quick screening. Therefore, both tests could be utilized practically and scientifically as part of a short, medium and long term planning process. Physical, functional and metabolic variables from these tests were shown to be valid (R^2^ = 0.850 - 0.904 [Marković et al., 2021]), reliable (Cronbach's Alpha = 0.721 - 0.958 and ICC = 0.779 - 0.977 [Marković et al., 2017, 2019, 2021]), and have limited sensitivity (*p* = 0.000 - 0.041 [Marković et al., 2018a, 2018b]). Due to the limited sensitivity of the results of previous research (Marković et al., 2018a, 2018b), i.e. observations on only two groups, there was a need to further determine the sensitivity of the tests.

Therefore, the aim of this study was to determine the differences in performance, cardiac, and metabolic variables of combat-specific fitness of wrestlers between athletes of varying sports levels. It was hypothesised that the SWFT and the SWPT would have high sensitivity (i.e., discriminative power).

## Methods

### Experimental Approach to the Problem

A cross-sectional experimental design was applied to assess the differences in the level of specific fitness of wrestlers of different sports levels.

### Participants

The sample consisted of 61 active and highly trained adult wrestlers of national and international levels from Serbia, from Greco-Roman and freestyle forms of wrestling, with at least 3 years of competitive experience. In relation to the competitive level, participants were allocated into three groups: national team (NT) wrestlers consisting of 15 athletes, first league (FL) competitors consisting of 20 athletes, and second league (SL) competitors consisting of 26 athletes. NT wrestlers competed internationally for the national team of the Republic of Serbia, FL wrestlers competed in the highest national league of the Republic of Serbia, while SL wrestlers competed in lower rank national competitions. The main characteristics of NT wrestlers were: age = 20.3 ± 2.7 years, body height (BH) = 169.6 ± 8.3 cm, body mass (BM) = 69.7 ± 7.7 kg, body mass index (BMI) = 24.16 ± 2.7 kg/m^2^, average competition experience = 11.0 ± 2.5 years, and training frequency = 7.7 ± 2.1 training sessions per week. The main characteristics of FL wrestlers were: age = 22.9 ± 4.3 years, BH = 172.8 ± 5.4 cm, BM = 76.7 ± 9.5 kg, BMI = 25.63 ± 2.4 kg/m^2^, average competition experience = 10.6 ± 4.2 years, training frequency = 6.5 ± 2.3 training sessions per week. The main characteristics of SL wrestlers were: age = 21.5 ± 2.8 years, BH = 180.2 ± 5.5 cm, BM = 86.9 ± 8.9 kg, BMI = 26.80 ± 3.1 kg/m^2^, average competition experience = 6.3 ± 2.4 years, training frequency = 5.69 ± 2.2 training sessions per week. The study was approved by the ethical board of the Faculty of Sport and Physical Education (484-2) and participants were thoroughly briefed about the tests that would be conducted and informed about the aim of the study. Only wrestlers who voluntarily agreed to be part of the study and signed a written informed consent form were included in the study. The research was carried out in accordance with the conditions of the Declaration of Helsinki, recommendations guiding physicians in biomedical research involving human subjects (World Medical Association, 2013).

### Design and Procedures

The process of data collection was performed in wrestling clubs, on a specific wrestling mat. All athletes were tested by the same testers and using pre-standardised measurement procedures (Marković et al., 2017, 2021). The tests were performed during the last week of the pre-competition mesocycle, whereby in the previous week they performed test polygons during the potentiation phase of a warm up in order to familiarise participants with the test. The tests were conducted during their regular training sessions. On the day of testing, all wrestlers went through the process of final theoretical and practical familiarisation with the tests. There were no specific requirements concerning diet or fluid intake before the test except to have a light breakfast a minimum of two hours prior to the test. Wrestlers were provided with sufficient drinking water at the test site. The test was preceded by a general warm-up lasting 10 min, and additional 5 min of a specific warm-up in the form of throwing a partner or a wrestling dummy, after which they rested for 5 min. During the test, participants performed a Suplex throw on a wrestling dummy (Suples, Ltd. ID, USA), whose weight was adjusted to the participants’ competing weight category. Participants whose BM was below 74.99 kg threw a 22 kg dummy; participants who were 75.00 - 89.99 kg threw a 27 kg dummy; and participants over 90.00 kg threw a 32 kg dummy (Marković et al., 2017). This way the relative load of the dummy was equalised between participants relative to their competing category. Indeed, wrestlers from the Greco-Roman style may perform Suplex technique more, but freestyle wrestlers perform it as well, just the risk of performing it is higher (i.e., the opponent can catch the legs) thus, they perform it less frequently in matches. Although it may not be often used, it is a complex routine highly effective in conditioning of wrestlers throughout the season. Therefore, wrestles from both styles are equally skilled in performing this technique.

The participants’ heart rates (HRs) were recorded as a functional measure of the cardiovascular system induced by an applied load. For this purpose, a H7 Polar Heart Rate Sensor (Polar, Inc., Lake Success, NY, USA) was used, which was placed around the participant’s chest before the test. Prior to fixing the strap to the chest, sensors were properly moistened. Once fixed to the chest, the tester checked if the HR sensor provided a stable signal. At the end of the test, wrestlers had one minute of active rest (walking and deep breathing on a wrestling mat), after which they took a sitting position, in order to collect blood lactate (La). The lactate concentration was analysed using a portable lactate analyser (Lactate Plus-NOVA biomedical, USA), using a lactate biosensor based on lactate oxidisation (Lactate Methodology **–** Lactate oxidase biosensor) (Hart et al., 2013; Kulandaivelan et al., 2009). The samples were collected from capillary blood, each time from a different finger by an experienced tester (Dopsaj and Janković, 2014; Marković et al., 2017). Blood samples were collected using a single-use lancet Unistik 3 Comfort (Owen Mumford Ltd. UK).

### Specific Wrestling Fitness Test

The SWFT was conducted following the previously explained procedure (Marković et al., 2017, 2018b, 2019, 2021). In short, the test consisted of three consecutive 30-s active periods, divided by 20 s of passive rest. Participants were instructed to complete as many maximal dummy throws as possible during each 30-s period. The final result was the total number of throws (Tn^Throws^) completed within the duration of the test.

### Specific Wrestling Performance Test

The test consisted of two 3-min rounds, which reflected the time of a round in a wrestling fight. The 3-min rounds were divided by 30 s of rest. The tasks within the first two minutes of both rounds were the same as was the third minute in both rounds (Table 1). In short, during the first 30 s participants performed a single dummy throw at the start of test time and then at the beginning of every 10 s. Then, they performed a maximal number of throws within 20 s, followed by 10 s of passive rest. The same sequence was repeated during the second minute and then, in the third minute, instead of 20 s of the maximal number of throws, participants performed 30 s of maximal throws. This was followed by a 30-s between-round passive rest. The second 3-min round was identical to the first one. The total number of throws executed in the phases of performing the maximum number of throws achieved during the entire test, i.e., both rounds, was taken as the final result of the test (Marković et al., 2017, 2018a, 2019, 2021).

**Table 1:** Structure of the SWPT

	Rounds				
Minutes	FIRST		Rest (s)	SECOND	
	Time intervals (s)	Number of throws		Time intervals (s)	Number of throws
1^st^ min	10	1	30	10	1
	10	1		10	1
	10	1		10	1
	**20**	Max. *		**20**	Max.*
	10	Rest		10	Rest
	
2^nd^ min	10	1		10	1
	10	1		10	1
	10	1		10	1
	**20**	Max.*		**20**	Max.*
	10	Rest		10	Rest
	
3^rd^ min	10	1		10	1
	10	1		10	1
	10	1		10	1
	**30**	Max.*		**30**	Max.*

* Throws which counted for the test result.

Considering the specificity and complexity of the techniques used in the aforementioned tests, the same tester controlled the correctness of actions of each participant during the tests in order to avoid interrater differences. The second tester controlled the timing and giving the instructions regarding the active and passive phases throughout the tests. The third tester was required to collect the HR data of each participant at the correct time. The testers verbally supported participants during the test in order to maximise their motivation and extract the maximum effort.

### Variables

The results of each test were presented as absolute performance measures (i.e., the number of throws), measures of cardiac response (i.e., HR) and metabolic response (La) or all integrated into index measures. The main performance measure on each test was a total number of throws (Tn^Throws^). The main measures of cardiac response to the applied effort were the HR at the end of the test (HR^0min^) and after the first minute of the rest interval (HR^1min^) expressed in beats per minute (bpm). The La concentration was collected in the third (La^3min^) and the fifth (La^5min^) minute of recovery as an indicator of post-effort metabolic acidosis (Åstrand et al., 2003), expressed in mmol/L. The Tn^Throws^, HR^0min^ and HR^1min^ were integrated into performance-specific cardiac response called a judo fitness index (SJF^Index^) developed by Franchini et al. (1998), whereby SJF^Index^ = (HR^0min^ + HR^1min^) / Tn^Throws^. The Tn^Throws^, HR^0min^, HR^1min^, La^3min^ and La^5min^ were integrated into performance-specific cardio-metabolic response called a wrestling performance index (SWP^Index^), developed by Marković et al. (2017), whereby SWP^Index^ = **((**HR^0min^ + HR^1min^) / (La^3min^ + La^5min^)) * Tn^Throws^. Thus, both the SWFT and the SWPT were defined through seven variables: Tn^Throws^, HR^0min^, HR^1min^, La^3min^, La^5min^, SJF^Index^, SWP^Index^.

### Statistical analyses

All analyses were carried out using the statistical package for social sciences (IBM, SPSS 20.0). The presented results include mean, standard deviation (SD) minimum (Min.) and maximum (Max.). Between-group differences were determined using multiple analysis of variance (MANOVA), while the least significant difference (Bonferroni) post-hoc was used to calculate the difference between individual groups. The level of significance was set at *p* < 0.05 (Hair et al., 1998). Cohen’s effect sizes (*d*) were calculated as the ratio of difference in mean scores to standard deviation. The following formula was used: *d* = (M2 - M1) / SD, where M1 and M2 were the means of the groups investigated and the SD was a pooled standard deviation of compared groups. The magnitude of the effects was defined as follows: small = 0.20 - 0.59, moderate = 0.60 - 1.19, large = 1.20 - 1.99 and very large ≥ 2.0 (Sullivan and Feinn, 2012). The factor analysis was used to determine the structure and the set of relationships between the original metabolic, cardiac, as well as the test results with the defined indexes of wrestling performance as proof of belonging to the commonly measured variance.

## Results

Table 2 shows the descriptive values for each variable adjusted to the sports level. Significant differences were obtained between wrestlers of different sports levels in Tn^Throws^ (F = 77.491, *p* < 0.001), SJF^Index^ (F = 49.710, *p* < 0.001), and SWP^Index^ (F = 31.205, *p* < 0.001) in the SWFT and in Tn^Throws^ (F = 71.051, *p* < 0.001), SJF^Index^ F = 45.343, *p* < 0.001), and SWP^Index^ (F = 26.820, *p* < 0.001) in the SWPT.

**Table 2:** Descriptive statistics for wrestlers of three different sports levels.

Variables		Sports level	Specific Wrestling	Fitness Test	Specific Wrestling	Performance Test
			Mean±SD	Min.-Max.	Mean±SD	Min.-Max.
Basic variables	**Tn^Throws^ (No)**	**National team**	32.40±1.8	29-35	47.13±3.3	41-53
		**First league**	28.30±1.7	26-33	41.65±4.2	35-50
		**Second league**	23.04±3.2	18-31	31.31±4.5	24-41
	**HR^0min^ (b/min)**	**National team**	185.67±14.1	171-218	186.13±11.6	174-211
		**First league**	183.90±6.9	172-200	185.50±7.4	168-195
		**Second league**	184.77±6.9	170-198	185.58±6.7	174-196
	**HR^1min^ (b/min)**	**National team**	166.40±16.0	131-195	165.53±9.2	143-180
		**First league**	166.65±8.6	153-184	168.60±9.1	145-184
		**Second league**	163.85±12.2	137-182	168.65±9.4	153-184
	**La^3min^ (mmol/l)**	**National team**	13.40±2.0	11-17	12.57±2.5	9-17
		**First league**	13.90±2.5	10-20	13.06±2.0	10-17.5
		**Second league**	13.36±2.0	9-16	12.86±2.4	8.4-17.1
	**La^5min^ (mmol/l)**	**National team**	14.40±2.0	12-19	12.21±2.5	7-17
		**First league**	13.80±2.8	10-19	12.43±2.3	9.6-17.4
		**Second league**	13.38±1.7	9-17	12.45±2.3	7.4-16
Integrated	**SJF^Index^ (Index value)**	**National team**	10.88±0.9	9.7-12.3	7.48±0.5	6.6-8.7
		**First league**	12.43±0.9	11.0-13.7	8.59±1.0	7.0-10.7
		**Second league**	15.40±2.1	11.0-19.3	11.56±1.9	8.3-15.5
	**SWP^Index^ (Index value)**	**National team**	413.44±62.0	337.2-513.2	691.56±124.8	477.9-906.6
		**First league**	366.85±50.4	269.3-446.3	585.52±67.7	447.5-698.5
		**Second league**	301.63±37.5	192.5-358.4	450.99±96.7	266.4-682.5

**Figure 1:**
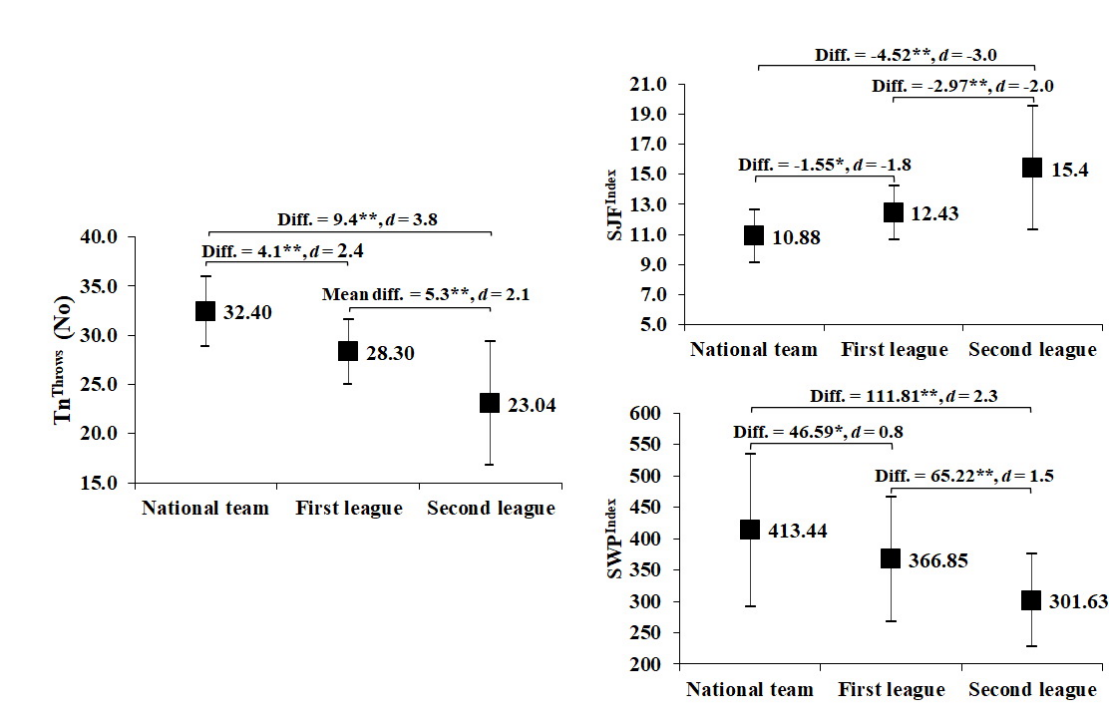
Pairwise comparison for the SWFT between wrestlers of different sports levels. Diff. – mean difference, *Significant at p < 0.05, **Significant at p < 0.001

**Figure 2:**
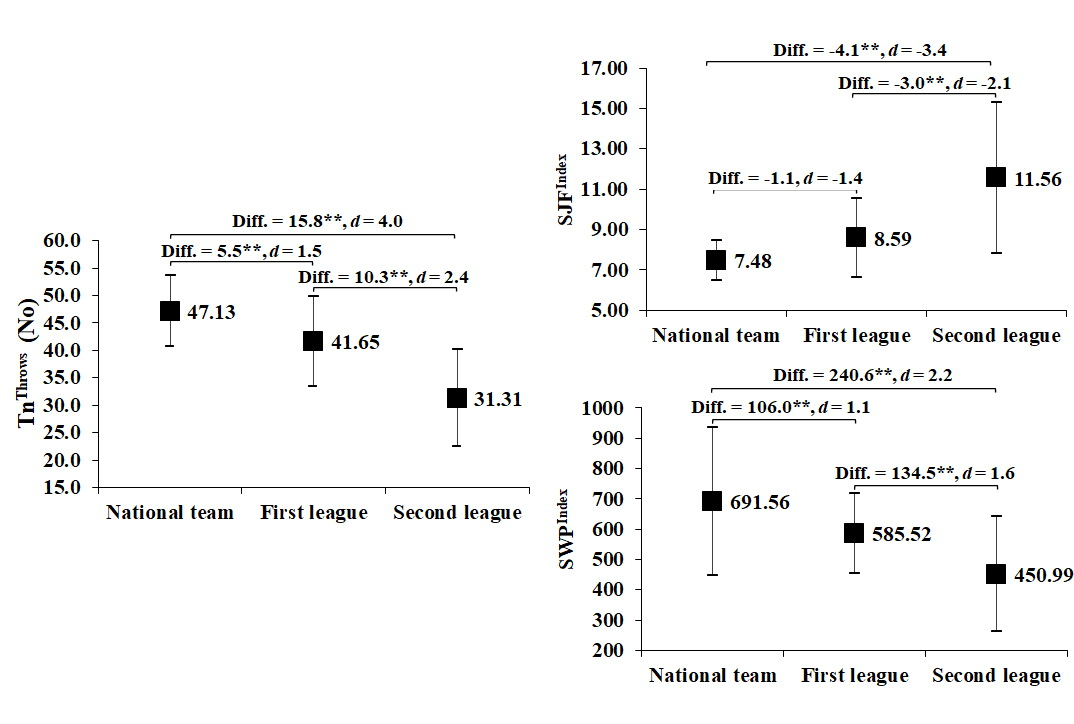
Pairwise comparison for the SWPT between wrestlers of different sports levels. Diff. – mean difference, *Significant at p < 0.05, **Significant at p < 0.001.

Pairwise comparison for the SWFT (Figure 1) and the SWPT (Figure 2) showed significant differences between NT and FL wrestlers, NT and SL wrestlers as well as between FL and SL wrestlers in Tn^Throws^, SJF^Index^, and SWP^Index^. The differences in performance for both tests were large to very large between NT and FL wrestlers, very large between NT and SL wrestlers, and large to very large between FL and SL wrestlers. The largest effect sizes occurred in Tn^Throws^, with the SWPT providing larger effect sizes.

Based on the measure sampling adequacy (Kaiser-Meyer-Olkin test; SWFT = 0.419, SWPT = 0.557) and the regularity of the multivariate space distribution (Bartlett’s Test of Sphericity, *p* = 0.000), it was determined that the results of the tested variables and their total variability could be reliably accepted for multivariate statistical analysis. Based on the calculated values of communalities of variables, it was determined that all variables were highly extracted into the common measuring space at the level from 0.863 (86.3%) for La^5min^ to 0.994 (99.4%) for Tn^Throws^ in the SWFT, and from 0.879 (87.9%) for HR^1min^ to 0.991 (99.1%) for Tn^Throws^ in the SWPT. Factor analysis showed that 93.24% and 94.59% of the common variance in the SWFT and the SWPT, respectively, could be explained by variables used, whereby three independent factors were extracted in both tests. The first factor explained 37.03% and 43.81% of the variance in the SWFT and the SWPT, respectively. The second factor explained 36.12% and 29.71% of the variance in the SWFT and the SWPT, respectively, whereas the third factor explained 20.10% and 21.07% of the variance in the SWFT and the SWPT, respectively. In both tests, the first factor was defined by three performance variables. The second factor was defined by two metabolic variables, while the third factor was defined by two cardiac response indicators (Table 3).

**Table 3:** Results of the defined matrix structure – the most significant factors.

Tests:	SWFT			SWPT		
Component:	1	2	3	1	2	3
**Tn^Throws^**	0.977			-0.982		
**SJF^Index^**	-0.957			0.971		
**SWP^Index^**	0.840			-0.811		
**La^5min^**		0.921			0.969	
**La^3min^**		0.919			0.954	
**HR^0min^**			0.954			0.951
**HR^1min^**			0.922			0.909

## Discussion

This study investigated the sensitivity of the SWFT and the SWPT in the evaluation of performance, cardiac, and metabolic variables of combat-specific fitness of wrestlers. The main findings showed that both tests determined significant differences between wrestlers of different sports levels, whereby wrestlers of a higher sports level performed significantly better than those of lower levels. Although the absolute measures of cardiac and metabolic responses were not different between sports levels, when integrated into performance indexes they provided a significant difference in performance-specific (i.e., combat-specific) cardiac-metabolic fitness of wrestlers. Thus, both tests showed high sensitivity in determination of wrestlers’ fitness, proving that the hypothesis of this study was true.

National team athletes who had a higher level of fitness, realised a higher number of Tn^Throws^ in both tests, compared with wrestlers of a lower competition level. Also, FL wrestlers from executed a significantly higher number of Tn^Throws^ than SL wrestlers in both tests, which confirms the results from previous research (Marković et al., 2018a, 2018b). The results obtained in absolute measures of cardiac and metabolic response of athletes considering a given effort were similar to those of Karnincic et al. (2009). They investigated La concentration during a wrestling match in national team competitors and club-level wrestlers of the same weight category and did not find a significant difference between groups, which was also the case in the current study. Similar results were found in judokas as elite athletes performed better in specific judo fitness tests than non-elite athletes, even though their cardiac and metabolic response did not differ (Franchini et al., 2005). Thus, absolute measures of the HR and La could not be considered as an accurate measure of physiological response to effort as they do not reflect the effectiveness and efficacy of the wrestler’s effort. Instead, cardio-metabolic response needs to be adjusted to attained performance as greater performance for the same cardio-metabolic response reflects better physical fitness.

To that end, significant differences occurred in the SJF^Index^ and SWP^Index^ which integrate cardio-metabolic response with wrestler's specific performance. SL wrestlers attained significantly worse results in these two indexes than FL and NT wrestlers in both tests. Moreover, FL competitors had lower results compared to NT competitors. This means that for the same heart rate wrestlers of a higher-level delivered larger amounts of oxygen to working muscles (i.e., increased capacity due to central adaptation), while for the same La concentration they performed more repetitions within a given time (i.e., higher muscle contraction intensity due to peripheral adaptation). More importantly, the SWPT and the SWFT provide not only highly valid and reliable (Marković et al., 2017, 2019, 2021), but also sensitive indicators of specific wrestlers fitness. In addition, factor analysis confirmed that the SWFT and the SWPT independently evaluate wrestler’s performance, cardiac, and metabolic responses. It is of note that Tn^Throws^ (i.e., performance-only) as well as the SJF^Index^ and SWP^Index^ (i.e., performance with cardio-metabolic response) showed high sensitivity in distinguishing between wrestlers of different sports levels. Therefore, the SWFT and the SWPT could be used with or without HR and La monitoring. This is of importance given that HR and La monitoring is rarely available in training settings as it may be costly, time consuming, and requires proper training. Considering this and the sample characteristics, initial cut-off values could be proposed (Table 4).

**Table 4:** Normative values

Evaluation:	Specific Wrestling Fitness Test			Specific Wrestling Performance Test		
	Tn^Throws^	SJF^Index^	SWP^Index^	Tn^Throws^	SJF^Index^	SWP^Index^
**Superior**	35 ≥	≤ 9.6	450.1 ≥	51 ≥	≤ 6.1	759.1 ≥
**Excellent**	33 - 34	9.7 - 10.8	416.9 - 450.0	47 - 50	6.2 - 7.3	690.9 - 759.0
**Very good**	30 - 32	10.9 - 12.0	383.8 - 416.8	43 - 46	7.4 - 8.4	622.6 - 690.8
**Good**	25 - 29	12.1 - 14.5	317.3 - 383.7	35 - 42	8.5 - 10.7	486.0 - 622.5
**Poor**	23 - 24	14.6 - 15.8	284.2 - 317.2	31 - 34	10.8 - 11.8	417.8 - 485.9
**Very poor**	20 - 22	15.9 - 17.0	251.0 - 284.1	27 - 30	11.9 - 12.9	349.5 - 417.7
**Bad**	≤ 19	17.1 ≥	≤ 250.9	≤ 26	13.0 ≥	≤ 349.4

Despite the established metric characteristics of the tests, in order to apply the tests, it is necessary to create norms as initial values for the comparison of the achieved results. By defining the most sensitive variables that assess specific wrestling fitness, Table 4 presents the seven-level normative values created in relation to the included sample, and as a function of both tests.

A few limitations should be pointed out before the final conclusion. The same set of studies, validity, reliability and sensitivity, should be replicated on all male and female wrestling weight categories and should also include some younger-age categories such as cadets and juniors. The study could be additionally conducted during the competitive season, as it would provide a clear overview on how a wrestler’s performance and cardiac and metabolic profiles should look like across the season. Moreover, it would allow more precise modelling and prediction of performance for the main competitions.

## Conclusions

Based on the results, it could be concluded that the SWFT and the SWPT provide very good sensitivity in the evaluation of physical fitness of wrestlers. Tn^Throws^ obtained in both tests was the most sensitive indicator of wrestling performance, clearly showing the difference between wrestlers of different sports levels. Therefore, even without collecting the HR and La concentration data, the tests provide a valuable insight into a wrestlers' current performance level. However, collecting HR and La concentration data improves the analysis as it provides cardio-metabolic response to a given performance, which is of importance for tapering of wrestlers' fitness during the season and fine tuning for specific matches. Coaches and performance analysts could use the information from these tests to evaluate the overall wrestler’s performance periodically so they could adjust the training programme as needed. Setting up the reference values for each part of the season may be of great value for the control and management of the training programme.
